# A Protein in the Yeast *Saccharomyces cerevisiae* Presents DNA Binding Homology to the p53 Checkpoint Protein and Tumor Suppressor

**DOI:** 10.3390/biom10030417

**Published:** 2020-03-07

**Authors:** Kanwal Farooqi, Marjan Ghazvini, Leah D. Pride, Louis Mazzella, David White, Ajay Pramanik, Jill Bargonetti, Carol Wood Moore

**Affiliations:** 1 Department of Molecular, Cellular and Biomedical Studies, City University of New York School of Medicine and B.S.-M.D. Program, Harris Hall, 160 Convent Avenue, New York, NY 10031, USA; kmf203@njms.rutgers.edu (K.F.); ghazvinm@sunyocc.edu (M.G.); lprideotubanjo@schools.nyc.gov (L.D.P.); ljmazzella@aol.com (L.M.); pramanik@sci.ccny.cuny.edu (A.P.); 2 City University of New York Graduate Center, Programs in Biochemistry and Biology, 365 Fifth Ave, New York, NY 10016, USA; davidwhite8828@gmail.com (D.W.); bargonetti@genectr.hunter.cuny.edu (J.B.); 3 Department of Biology, Hunter College, City University of New York, 695 Park Avenue, New York, NY 10021, USA

**Keywords:** p53 response elements, yeast, SCS, MDM2, transactivation, p53 DNA binding sites, p53 binding site factor, *Saccharomyes cerevisiae*

## Abstract

*Saccharomyces cerevisiae* does not contain a p53 homolog. Utilizing this yeast as an in vivo test tube model, our aim was to investigate if a yeast protein would show p53 DNA binding homology. Electrophoretic mobility shift analyses revealed the formation of specific DNA-protein complexes consisting of *S. cerevisiae* nuclear protein(s) and oligonucleotides containing p53 DNA binding sites. A *S. cerevisiae* p53 binding site factor (Scp53BSF) bound to a p53 synthetic DNA-consensus sequence (SCS) and a p53 binding-site sequence from the *MDM2* oncogene. The complexes were of comparable size. Like mammalian p53, the affinity of Scp53BSF for the SCS oligonucleotide was higher than for the MDM2 oligonucleotide. Binding of Scp53BSF to the SCS and MDM2 oligonucleotides was strongly competed by unlabeled oligonucleotides containing mammalian p53 sites, but very little by a mutated site oligonucleotide. Importantly, Scp53BSF-DNA binding activity was significantly induced in extracts from cells with DNA damage. This resulted in dose-dependent coordinated activation of transcription when using p53-binding site reporter constructs. An ancient p53-like DNA binding protein may have been found, and activation of DNA-associated factors to p53 response elements may have functions not yet determined.

## 1. Introduction

The evaluation of novel chemotherapeutics that target multiple coordinated signal transduction programs is challenged by the genetic heterogeneity of cancer and the lack of an association of critical biomarkers for chemotherapeutic efficacy. While multiple genetic mutations are found in each subtype of human cancer, the gene found mutated most often is p53 [1,2,3]. In normal cells, the p53 checkpoint protein maintains genomic stability, transcription activation, and tumor suppression. In more than 50% of human tumors, the p53 tumor suppressor is mutated or lost [3,4,5]. In tumors that do not have mutant p53, other mutations changing the underlying p53 biology of the cell often exist. Many clinically-used chemotherapeutics cause DNA damage that activates wild-type p53. In normal cells, wild-type p53 is present at low levels. However, following DNA damage, kinase cascades are activated causing phosphorylation and stabilization of the p53 protein. The p53 protein then activates the transcription of a cohort of down-stream target genes that work to initiate varying outcomes, such as cell cycle arrest, autophagy, senescence, metabolic processes and apoptosis [1].

Oncogenic mutant p53, on the other hand, is present in high levels in tumor cells and is unable to activate a downstream signal transduction pathway following DNA damage. Many p53 mutations have been associated with gain-of-function oncogenic activity, not simply loss-of-function [3]. Many of these mutant p53 proteins acquire oncogenic properties that enable them to promote invasion, metastasis, proliferation and cell survival [6,7,8]. While wild-type p53 functions as a DNA damage sensor protein, oncogenic mutants lack this function and facilitate genomic instability [9,10,11]. A greater understanding of what types of signaling networks perform well in either p53 deficient cells, or in cells with oncogenic mutant p53, is an important focus for the next generation of cancer medicines to treat individual patients.

The budding yeast, *Saccharomyces cerevisiae*, does not express endogenous p53. As a result, *S. cerevisiae* has been used as an in vivo test tube for examining the DNA-protein interactions of wild-type and oncogenic mutant p53 [12,13,14,15,16,17,18,19,20]. Human p53 and its family members can activate transcription in yeast from a synthetic consensus p53 responsive element (RE) exactly fitting the model of two decameric half-sites separated by 0–13 nucleotides (nt), originally defined by the consensus RRRCWWGYYY (n = 0–13) RRRCWWGYYY, as well as many variants of the p53 RE [16,21]. Large scale genome-wide sequence analyses indicate that numerous model and variant p53 RE sequences reside within the yeast genome (Leah D. Pride, unpublished results).

Human p53 inhibits growth in the fission yeast *Schizosaccharomyces pombe* [22,23]. In *S. cerevisiae*, numerous mechanisms of p53 regulation have been elucidated. For example, human p53 activates transcription of reporter genes placed under the control of hybrid promoters containing p53-binding sequences, and a gene reporter system involving a color change can be used as a readout for p53 activity [14,16,21,24,25,26,27]. Mdm2-dependent post-translational modifications of p53 affect p53 localization and function [28]. In both budding yeast and human cell systems, the organization and sequence of target response elements (REs) and the levels of p53 influence p53-mediated transactivation [13,15,21,26,29,30,31,32,33,34]. Coactivator requirements for p53-dependent transcription in yeast revealed exceptional similarities with those in mammalian cells [13,35,36]. Thus, the yeast model system can be a useful in vivo model system to dissect the accessory components that potentiate the p53 network [16,17,21,26,34,36,37,38]. However, because yeast lacks endogenous p53, it can also be used to elucidate p53-independent activation of canonical p53 target genes.

For the current studies, we used natural and synthetic consensus DNA sequences for p53 to examine DNA-binding activities of *S. cerevisiae* nuclear extracts to oligonucleotides containing p53 DNA binding sites. DNA damage is known to activate p53 DNA binding activity, and therefore we tested if yeast contained a proteomic structural homolog that would also respond to the stress of DNA damage. The clinical importance of addressing this question is that if the ancient DNA damage response can be seen to contain a protein with functional, but not DNA sequence, homology to p53, then this can be used to understand important functional properties of this critical tumor suppressor pathway. We further investigated the potential of yeast to activate transcription from p53 REs after treatments with the chemotherapeutic and DNA-damaging bleomycin-phleomycin drug class. Cellular activities of these oxidative DNA-cleaving drugs are considered radiomimetic since they exhibit similarities with ionizing radiation [39,40,41,42,43]. We examined if drug-induced DNA damage activated binding of a protein to p53 REs, thus identifying factors involved in DNA-damage signal transduction in yeast. We observed that DNA damage activated the binding of an endogenous yeast protein, or protein complex, to p53 REs. We also observed coordinated activation of transcription when using yeast reporter constructs.

## 2. Materials and Methods

### 2.1. Strains and Culturing Conditions

The wild-type yeast strain, CM-1293 (*Mat*a/*Mat*α*, ade2-119/ade2-40, trp5-12/trp5-27, ilv1-92/ilv1-92, cyc1-131/cyc1-45*), was grown with aeration in non-synthetic complete liquid medium (1% yeast extract, 2% peptone, 2% glucose, 2% agar) supplemented with 0.008% adenine sulfate (YPAD) at 30 °C [42,43,44]. Precultures were inoculated with a single colony from a YPAD plate and grown at 30 °C to approximately 2.5 × 10^8^ cells/mL. For each experiment, initial titers of 5 × 10^6^ cells/mL were inoculated from fresh precultures and grown to approximately 2 × 10^8^ cells/mL. Cells were harvested at 4 °C by centrifugation in a Sorvall RC5C centrifuge at 5000 rpm for 5 min and washed three times in deionized water.

### 2.2. Nuclear Preparations

The established method of Dunn and Wobbe [45] was used for making nuclear preparations. Washed pellets of each population of untreated and treated cells were resuspended in 750 μL of buffer containing 5% 1 M Tris-HCl (pH 7.5), 1% 1 M MgCl_2_, 50% 2 M sorbitol, 3% 1 M dithiothreitol (DTT) and 2 μg/mL of leupeptin, and incubated for 15 min at 4 °C. The suspension was vortexed and centrifuged for 5 min at 2000 rpm at 4 °C in a Sorvall RCB 6000T rotor. The pellet was resuspended in 2.25 mL of the same buffer, with lyticase (Sigma-Aldrich, St. Louis, MO, USA) added to 1245 units/mL. The solution was incubated while shaking at 3000 rpm at 30 °C. The conversion to spheroplasts was monitored under a microscope. Spheroplasts were centrifuged at 2000 rpm at 4 °C for 5 min in a Sorvall RT6000B centrifuge, and the pellet was washed two times with the buffer. After every wash, the solution was vortexed and centrifuged at 2000 rpm for 5 min at 4 °C. The final pellet was resuspended in 1.13 mL of buffer. The suspension was transferred drop by drop into 25 mL of ice-cold Ficoll solution (50% of 36% Ficoll, 1% 1 M Tris-HCl [pH 7.5], 2% 1 M KCl, 0.5% 1 M MgCl_2_, 0.3% 1 M DTT, 0.4% 250 mM EDTA, 0.1% 1 M phenylmethylsulfonyl fluoride [PMSF], and 2 μg/mL leupeptin). This solution was kept at 4 °C with constant stirring. The Ficoll solution was transferred into centrifuge tubes and pelleted for 5 min at 6500 rpm at 4 °C in a Sorvall RC5C SS-34 rotor. The supernatant was centrifuged again at 16,000 rpm at 4 °C for 20 min in the same rotor, and the resulting pellet was stored at −80 °C.

### 2.3. Extraction of Proteins

The protocol for extracting proteins from nuclear preparations was adapted from published procedures [46,47]. The pellet was resuspended in 3 mL of nuclear lysis buffer (4% Hepes 1 M pH 7.9, 50% glycerol 100%, 16.8% NaCl 5 M, 0.3% MgCl_2_ 1 M, 0.08% EDTA 0.5 M pH 8.0, 0.4% PMSF 1 M, 0.1% DTT 1 M, and 2 μg/mL leupeptin that had been previously dissolved in this buffer) at 4 °C. Yeast nuclei were disrupted by 10 passes of each sample through a 26 gauge needle. The extracts were shaken at 4 °C on a VWR S/P rotator (Bridgeport, NJ, USA) at 220 rpm for 30 min. The protein extracts were isolated as supernatants by centrifugation at 17,000 rpm for 30 min at 4 °C in an RCB 6000T rotor. The concentration of proteins in each extract was determined by Bradford protein analysis using a Bio-Rad Laboratories (Hercules, CA, USA) protein assay kit [48].

### 2.4. Sodium Dodecyl Sulphate-polyacrylamide Gel Electrophoresis (SDS-PAGE)

SDS-polyacrylamide gels were prepared as described by Gallagher [49]. The separating gel contained 10% acrylamide (30% acrylamide/0.8% bisacrylamide), 25% 4× Tris-HCl/SDS pH 8.8, 0.33% 10% (*w*/*v*) ammonium persulfate, and 0.066% *N,N,N’,N’*-tetramethylethylenediamine (TEMED). The stacking gel contained 3.9% acrylamide (30% acrylamide/0.8% bisacrylamide), 25% 4× Tris-HCl/SDS pH 6.8, 0.5% of 10% (*w*/*v*) ammonium persulfate, and 0.1% TEMED. All solutions were prepared and stored at 4 °C. The protean-II SDS-PAGE system from Bio-Rad was used to separate the proteins in the extracts. Before loading the samples, the protein gel was pre-run for 20 min at 25 mA. Equal concentrations of protein were loaded in each lane on the gel, and run at 13 °C for 2 h at 25 mA through the stacking gel and 5 h at 35 mA through the separating gel. Bio-Rad broad range standards were run to calculate the molecular weights of the proteins [49,50]. The gel was fixed in a fixative enhancer solution (Bio-Rad Silver Stain Plus Kit) for 24 h, washed with deionized water twice for 10 min, and stained with the silver stain reagent provided in the Bio-Rad Silver Stain Plus Kit. The staining was stopped in 5% acetic acid.

### 2.5. Oligonucleotides and DNA Labeling

The synthetic oligonucleotides used in this study were purchased from Integrated DNA Technologies (Coralville, IA, USA) or Operon Technologies (Alameda, CA, USA). Labeling of the oligonucleotides was performed with the large fragment of DNA polymerase (New England BioLabs, Beverly, MA, USA) and [^32^P]dCTP (Perkin Elmer Life Sciences, Boston, MA, USA) as described previously [51]. The oligonucleotides [52] were as follows:

1. Human p53 super consensus sequence (SCS):
  Top: 5′-TCG AGC CGG GCA TGT CCG GGC ATG TCC GGG CAT GTC-3′
  Bottom: 5′-TCG AGA CAT GCC CGG ACA TGC CCG GAC ATG CCC GGC-3′
2. The p53 binding site sequence from the *MDM2* oncogene:
  Top: 5′-GAT CCC TGG TCA AGT TGG GAC ACG TCC GGC GTC GGC TGT CGG AGG AGC TAA GTC CTG ACA TGT CTCCG-3′
  Bottom: 5′-GAT CCG GAG ACA TGT CAG GAC TTA CCT CCT CCG ACA GCC GAC GCC GGA CGT GTC CCA ACT TGA CCAGG-3′
3. Mutant RGC:
  Top: 5′-TCG AGT TTA ATG GAC TTT AAT GGC CTT TAA TTTTC-3′
  Bottom: 5′-TCG AGA AAA TTA AAG GCC ATT AAA GTC CAT TAAAC-3′

### 2.6. Electrophoretic Mobility Shift Assays (EMSAs)

EMSA experiments were carried out in reaction mixtures (30 µL) with 0.4 pmole of radiolabeled oligonucleotide. Variable amounts of protein extracts were added as indicated, and the reaction was incubated for 20 min at room temperature in a reaction buffer containing 20 mM Hepes, pH 7.8, 100 mM KCl, 1 mM EDTA (pH 8.0), 1 mM DTT, 1 μg sheared salmon sperm DNA, and 10% glycerol. Competition assays were performed in the presence of radiolabeled oligonucleotide and unlabeled oligonucleotide. The protein-DNA complexes were resolved on 4% polyacrylamide gels via electrophoresis (gels were pre-run at 100 V for 1 h at 4 °C) at 200 V for 2.5 h. Gels were dried for 1.5 h at 60 °C, and autoradiography was performed.

### 2.7. Pulsed-field Gel Electrophoresis

Cells were treated with the anticancer formulation of bleomycin, Blenoxane, as previously described [42,43]. The drug was provided as a gift from Bristol Myers Squibb Laboratories through the courtesy of Dr. William T. Bradner, Ms. Linda Sanders and Mr. Daniel T. Elliot. Cells were incubated with aeration with or without the drug for various periods in deionized water at 1 to 2 × 10^7^ cells/mL. At the end of each treatment period, EDTA was added to 0.025 M prior to washing the cells three times with 0.05 M EDTA at 4 °C. We assessed genomic integrity by pulsed-field gel electrophoresis (PFGE) as previously described [43]. Because of a rigid cell wall, yeast cells survive for long periods in deionized water or buffer without lethal effects and without acquiring DNA breaks [43].

### 2.8. Gene Reporter Assays and Transactivation Experiments

Strains yAFM containing the P21-5′ RE and yAFM containing the Con-A RE were kindly provided by Drs. Michael Resnick and Alberto Inga [26,36] for the *ADE2*-transactivation assays [26,31,32,33,53,54,55,56]. The sequences of the REs are as follows:

Mammalian P21-5′ CAA CAT GTT GGG ACA TGT TC [26,57]

Artificial Con-A GGG CAT GTC CGG GCA TGT CC [26,58]

Preliminary experiments were carried out to determine which procedures best allowed the ade-requiring yAFM strains to grow and exhibit red or pink to white color changes, and whether colony, patch, pie or spot plating worked best. Because the strains require adenine to grow, they do not grow on minimal synthetic medium lacking adenine supplementation and grow red on the same medium with low amounts of adenine. After testing minimal and non-synthetic complete media containing varying amounts of adenine and phleomycin D1 (in the formulation of Zeocin from Invitrogen Company, Carlsbad, CA), we found the low amounts of adenine in yeast extract in non-synthetic complete YPD medium (YPAD without adenine) permitted the best growth and color distinctions. The pie method of applying and spreading the cells worked best in conjunction with this medium.

All experiments included control strains without REs. The adenine-requiring strains were CM1452-98B (*MAT*α *ura3-52 ade2-40 leu2-3 ilv1-92*) [59] and W303-1A (*MAT*α *ade2-1 can1-100 his3-11,15 leu2-3,112 trp1-1 ura3-1*) [60]. The strain prototrophic for adenine was CB80 (*MAT***a** *leu2-1 ura3-52 his3-200 trp1-3*) [61]. All plates were incubated for three days at 30 °C. YPD and YPG (1% yeast extract, 2% peptone, 3% *v*/*v* glycerol, and 2% agar) media were used to test for growth of colonies that may have lost mitochondrial function (petite colonies).

## 3. Results

### 3.1. A Nuclear Yeast Protein Binds Specifically to the p53 Consensus Sequence

**Figure 1:**
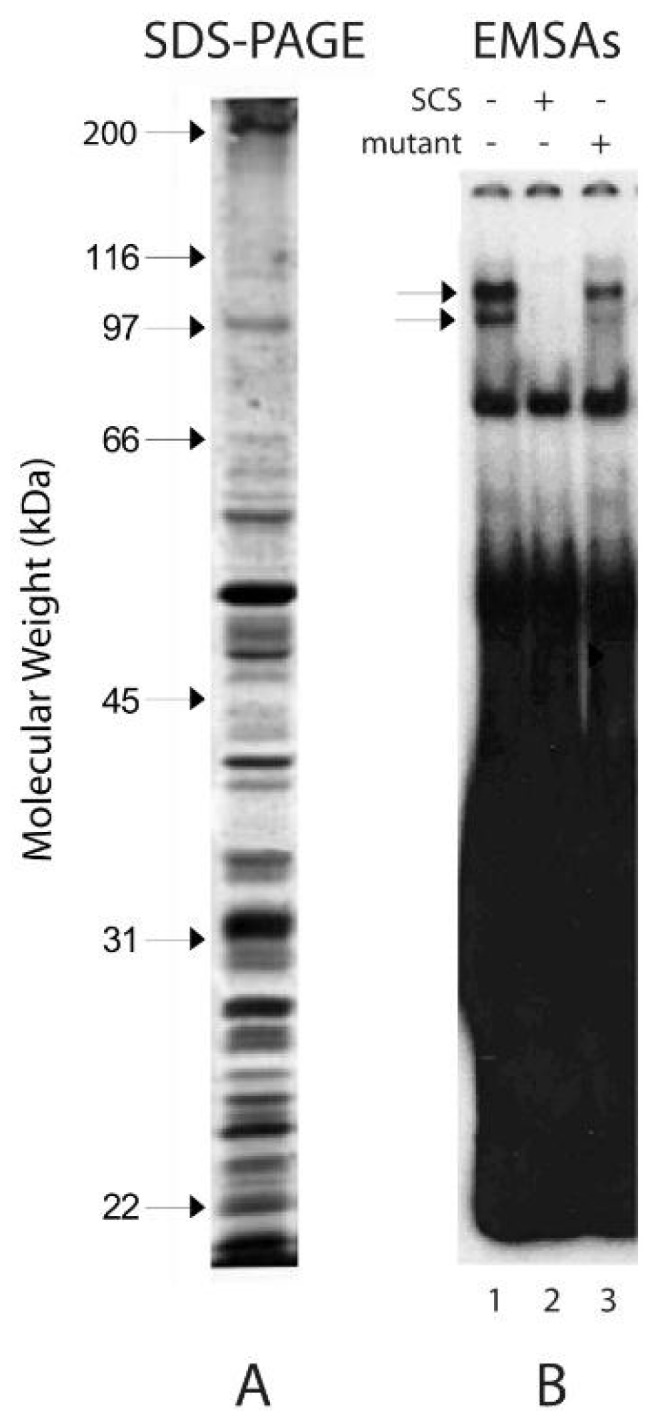
Yeast protein complexes bind specifically to the human p53 super consensus sequence. **A**. Proteins used in the gel shift assays were extracted from nuclear preparations from fresh cells grown to the early stationary phase in liquid YPAD medium. Sixty μg of protein were loaded and run on 10% SDS-PAGE gels. Molecular weights of nuclear proteins were determined by molecular weight standards run in adjacent lanes. **B**. Electrophoretic Mobility Shift Assays (EMSAs) of 5.1 μg of protein hybridized to the ^32^P-radiolabeled oligonucleotide (0.15 pmole) containing the p53 synthetic DNA-consensus sequence (SCS). Lane 1: no competitor. Lane 2: competition with 15 pmole (100x excess) of unlabeled p53 SCS oligonucleotide. Lane 3: competition with 15 pmole (100x excess) of unlabeled mutant RGC (mtRGC) oligonucleotide. The gel was run until the bromophenol blue dye reached two inches from the bottom of the chamber. Bands that contain proteins that specifically bound to DNA in the complexes are indicated with two arrows.

Nuclear extracts were examined for potential factors or complexes in early stationary-phase yeast that bound specifically to the human p53 super consensus sequence. The SCS oligonucleotide contains a sequence that acts as a super consensus DNA-binding site [62]. Proteins extracted from nuclear preparations and run on SDS-polyacrylamide gels are illustrated in Figure 1A. The relative amounts of each protein in the extracts were consistent among all protein extracts throughout the studies. Judged by the apparent constant molecular masses of the proteins in freshly prepared samples and in samples that had been repeatedly frozen and thawed, the integrity and stability of the proteins appeared to be preserved from multiple sample preparations and throughout several freeze-thaw steps.

Competition EMSAs were carried out to determine if any of the proteins in the extracts bound specifically to the p53 DNA super consensus site [47,52]. Competition was carried out with unlabeled SCS oligonucleotide and a common mutant oligonucleotide used in gel shifts, the mutant ribosomal gene cluster (mtRGC), mutated within the p53 recognition element so it no longer binds p53 protein [52]. Typical results from gel shift analyses with the radiolabeled SCS oligonucleotide and competition with the unlabeled SCS and mtRGC oligonucleotides are shown in Figure 1B. Two specific nuclear protein complexes bound strongly to the radiolabeled SCS oligonucleotide (Figure 1B, lane 1, as indicated by arrows). Binding in the two complexes was determined to be specific since it was competed efficiently in the presence of unlabeled SCS oligonucleotide (Figure 1B, lane 2), but not efficiently by the mtRGC oligonucleotide (Figure 1B, lane 3). The nonspecific DNA binding in complexes (those not labeled by the arrows) was not considered important for these studies. On the basis of the specificity of the DNA binding activity conferred by the yeast complexes and for simplicity in referring to this activity, we henceforth refer to it as Scp53BSF (*S. cerevisiae* p53 binding site factor).

### 3.2. Relative Strengths of Competitive Inhibition of SCS Binding by SCS, the Naturally Occurring p53 Binding Site in the MDM2 Gene, and Mutant RGC

**Figure 2:**
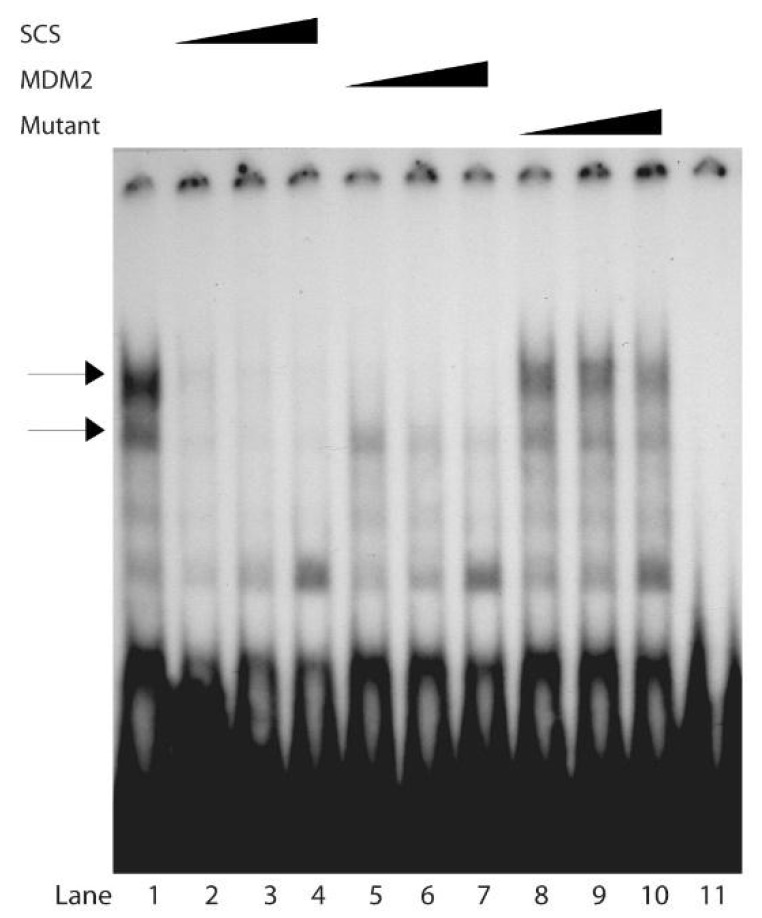
Competitive binding of the SCS-protein complexes with varying excesses of different oligonucleotides containing p53 binding sites. EMSAs in lanes 1–10 contained 4 μg of protein extract and 0.5 pmole of radiolabeled SCS. Lane 1: no competitor. Lanes 2–4: binding in the presence of excess non-radiolabeled SCS oligonucleotide. Lanes 5–7: binding in the presence of the natural p53 binding site from the *MDM2* gene. Lanes 8–10: binding in the presence of the mutant RGC (mtRGC) oligonucleotide. Competition was carried out with 40 fold (lanes 2, 5 and 8), 80 fold (lanes 3, 6, and 9), and 160 fold (lanes 4, 7, and 10) excesses of oligonucleotides. The two bands that are responsive to the competition are indicated by the two arrows. Lane 11 contains 0.5 pmole of radiolabeled SCS without protein or competitor. To increase resolving power, this gel was run until the bromophenol blue reached the bottom.

We next examined through competition experiments the relative affinities of the Scp53BSF complexes to the SCS, the wild-type p53-binding site from the *MDM2* gene, and mutant RGC. The human *MDM2* gene is transcriptionally regulated by p53 [63]. While Scp53BSF binding to the SCS was strong without competitor (Figure 2, lane 1, indicated by the two arrows), binding was almost completely inhibited by 40-, 80- and 160-fold excesses of unlabeled SCS (Figure 2, lanes 2–4). Similarly, the unlabeled MDM2 oligonucleotide competed with the yeast complexes bound to the SCS oligonucleotide (Figure 2, lanes 5–7), although MDM2 competed slightly less than the SCS. The competition by 40-, 80- or 160-fold excesses of the mutated oligonucleotide was quite low (lanes 9–11) in comparison to the SCS and MDM2 oligonucleotides. A faster migrating species increased in quantity with increasing amounts of all three unlabeled competitors. It is possible that Scp53BSF binds DNA as a tetramer, much like p53. The fastest form of Scp53BSF could be an increased occurrence of the monomer. The important observation when using a lower concentration of labeled oligonucleotide is that both unlabeled SCS and MDM2 oligonucleotides compete the binding far better than the mtRGC oligonucleotide.

### 3.3. Similar Size of the Yeast Complex When Bound to the SCS and Natural Mammalian p53 Specific MDM2 Sequence

**Figure 3:**
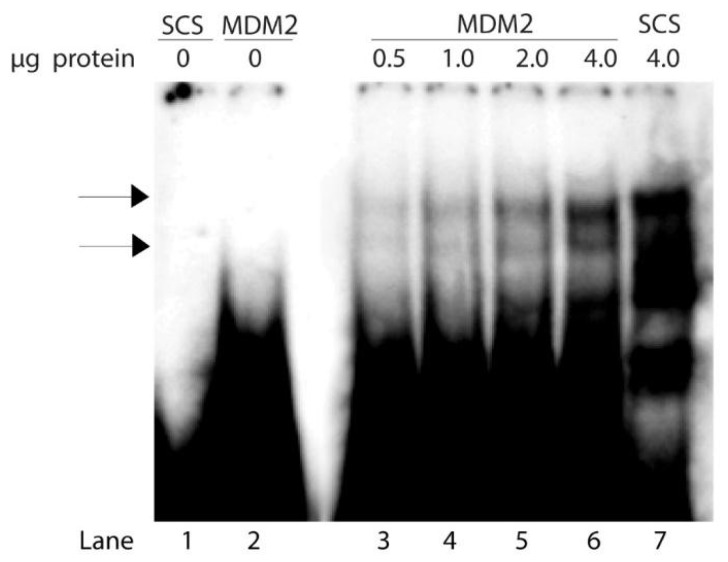
Increased binding of Scp53BSF to the natural p53 binding site from the *MDM2* gene as a function of increasing quantities of protein. Concentration-dependent binding was examined using 0.45 pmole of radiolabeled MDM2 oligonucleotide and 0.5 to 4 μg of protein from the nuclear extracts. Lane 1: radiolabeled SCS oligonucleotide, no protein. Lane 2: radiolabeled MDM2 oligonucleotide, no protein. Lane 3-6: radiolabeled MDM2 oligonucleotide with 0.5 μg (lane 3), 1 μg (lane 4), 2 μg (lane 5) and 4 μg (lane 6) of protein. Lane 7: radiolabeled SCS oligonucleotide with 4 μg of protein.

The ability of the MDM2 p53 binding site to compete with the binding of Scp53BSF complexes to the SCS oligonucleotide suggested that the complexes might also bind to radiolabeled MDM2 oligonucleotide. Thus, we analyzed the relative DNA binding affinity of Scp53BSF to the MDM2 oligonucleotide as a function of increasing quantities of yeast nuclear extract in the binding reactions. Figure 3 illustrates quantitative analyses of the binding activities of the complexes to the MDM2 oligonucleotide. Binding to the MDM2 oligonucleotide was detected with the addition of 0.5 μg protein, and doubling the amount of yeast protein to as high as 4 μg doubled the binding each time (Figure 3, lanes 3–6, complexes indicated by arrows). Binding affinities of the MDM2 and SCS oligonucleotides were compared at the highest protein concentration (4 μg), and the results indicate that SCS affinity is higher than MDM2 affinity (Figure 3, lane 7 versus lane 6). This was consistent with the increased competition demonstrated by the SCS oligonucleotide compared to the MDM2 oligonucleotide in Figure 2, and the higher binding affinity of mammalian p53 to the consensus sequence in comparison to the MDM2 sequence [47].

**Figure 4:**
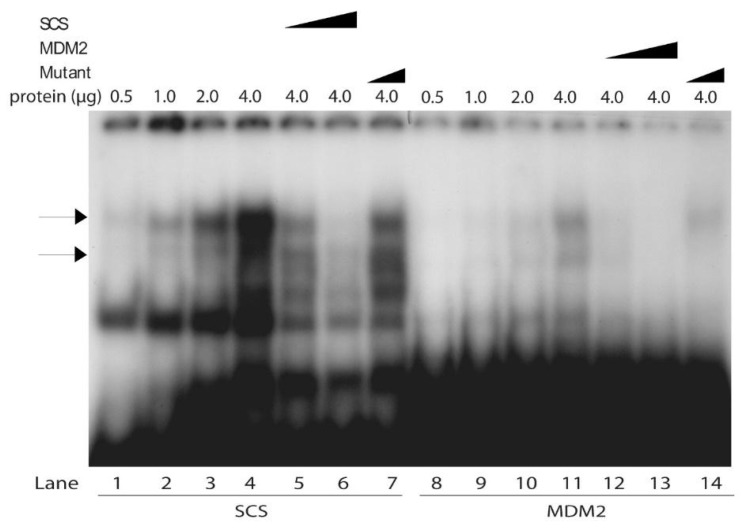
The Scp53BSF appears to be the same size when bound to the SCS or MDM2 oligonucleotide in comparative gel shift analyses. EMSA analyses were carried out with varying amounts of protein and 0.45 pmole of radiolabeled SCS (lanes 1–7) or wild-type MDM2 oligonucleotide (lanes 8–14). Competition was carried out with 4- or 40-fold excesses of SCS (lanes 5 and 6) and MDM2 (lanes 12 and 13) oligonucleotides, and 40-fold excess of mutant RGC oligonucleotide (lanes 7 and 14). Films were overexposed to maximize the resolution of the protein-MDM2 oligonucleotide complexes when low levels of protein were present or after competition with 40-fold excess MDM2. The two bands that appear to comprise the SCS- and MDM2 complexes are indicated with arrows.

Next, the mobilities of the DNA binding species of the yeast complexes to the SCS and MDM2 oligonucleotides were compared as a function of adding increasing amounts of yeast protein and different amounts of SCS, MDM2 and mutant oligonucleotide competitors. The amount of binding of Scp53BSF to both SCS and MDM2 oligonucleotides increased as a function of increasing protein concentrations (Figure 4, lanes 1–4 and 8–11, as indicated by the two arrows). While the MDM2 site had a lower affinity than SCS for Scp53BSF, the complexes on the MDM2 site appeared to be the same sizes as those on the SCS site. Interestingly, four species were observed. This was reminiscent of work with tetrameric p53 in EMSA assays [64]. The competition and specificity of Scp53BSF to the SCS and MDM2 sites are also shown in Figure 4 with non-labeled self oligonucleotides, and contrasted with the inefficient competition by mutant oligonucleotide (Figure 4, lanes 5–7 and 12–14).

### 3.4. Enhanced Stability of the SCS Oligonucleotide-protein Complexes After Oxidative DNA Damage

**Figure 5:**
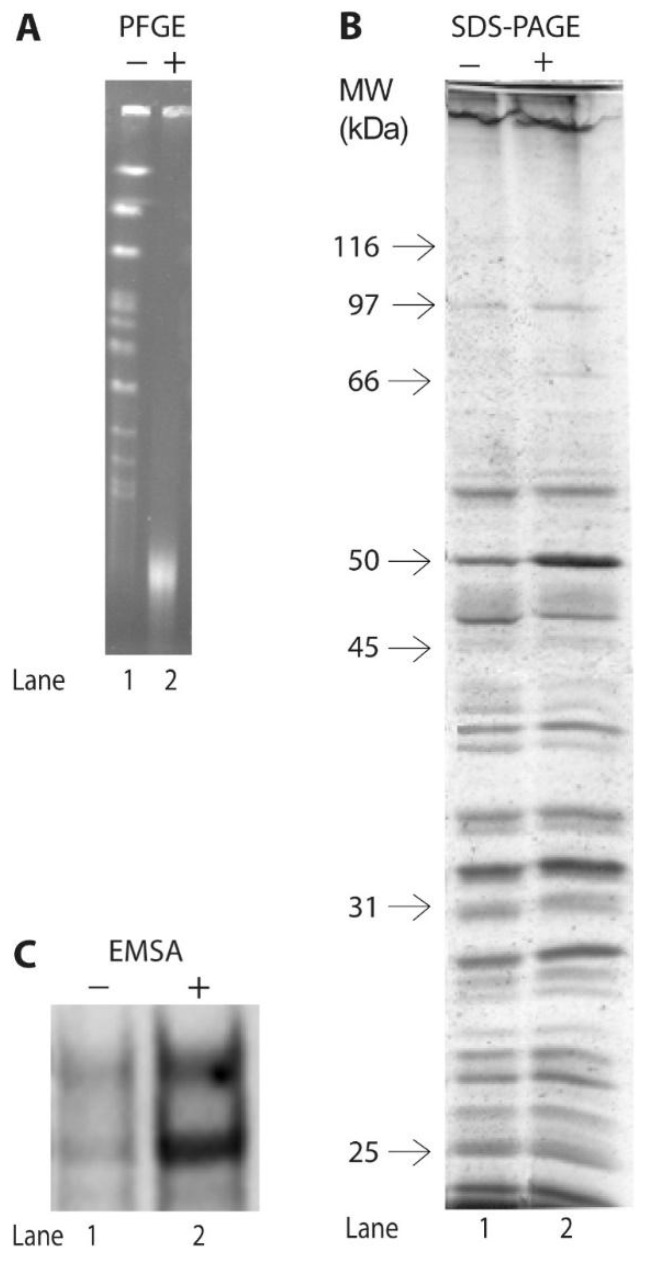
DNA damage, the integrity of proteins, and stability of the Scp53BSF-DNA complexes. Cells in the early stationary phase of growth were incubated with or without bleomycin for 24 h. **A**: pulsed-field gel electrophoresis (PFGE) illustrating integrity of chromosomes in cells incubated without bleomycin (−) and chromosomal destruction in cells incubated with 5 μg/mL bleomycin (+). Equivalent numbers of cells were loaded in each lane. **B**: The integrity of the proteins extracted from untreated and treated cells. Sixty μg of protein from nuclear preparations used for Figure 5A were loaded and run on a 10% SDS-PAGE gel. Molecular weights of nuclear proteins were determined by molecular weight standards run in adjacent lanes. Protein integrity was observed in all extracts from untreated and treated cells. **C**: Comparative EMSAs of 0.45 pmole of radiolabeled SCS, 40-fold excess unlabeled SCS oligonucleotide, and equivalent quantities of protein extracted from untreated cells (-) and 5 μg/mL bleomycin-treated cells (+). Only the two bands corresponding to the Scp53BSF-SCS complexes are shown.

We next determined the stability of the Scp53BSF-SCS complex after DNA damage. Yeast cells were incubated under non-growing conditions for 24 h with or without bleomycin. Chromosomal stability was assessed with PFGE, during which individual chromosomes separate into distinct bands according to molecular weight and electric field interaction [43]. Without bleomycin treatment, chromosomes were intact after the 24 h incubation (Figure 5A), and nuclear proteins extracted and analyzed by SDS-PAGE were intact (Figure 5B). After 24 h treatments, double-strand breaks in DNAs caused chromosomal degradation, and the degraded chromosomes either accumulated at the bottom of the gels as a diffuse smear or left the gels (Figure 5A). Proteins extracted from treated cells, on the other hand, remained intact (Figure 5B). The amounts and integrity of proteins extracted from untreated and treated cells appeared comparable (Figure 5B).

Oligonucleotide-binding activities of equivalent amounts of protein from untreated and treated cells were analyzed using radiolabeled SCS and unlabeled SCS. As shown in Figure 5C, the abundance of the Scp53BSF-SCS complexes in treated cells was significantly higher than their amounts in untreated cells. We interpret this result to indicate that either the DNA binding activity of the Scp53BSF is more stable after DNA damage than in untreated cells, or that the protein is post-translationally modified to produce a more active DNA binding species after DNA damage.

### 3.5. DNA-damage Inducible Regulation of Transactivation from Reporter Constructs

Finding a protein or protein complex that recognized and bound specifically to known p53 binding sites, and that it increased with DNA damage, suggested transcriptional activation of p53 binding site REs might be stimulated after treatments with DNA-damaging agents. To test this hypothesis, we turned to an in vivo system based and developed in *S. cerevisiae*. This system utilizes a reporter gene located at its native chromosomal position in a single copy and a tightly regulated promoter to compare transactivation activities of different p53 binding site REs [26,31,32,33,36,53,54,55,56,65]. Transcription of the reporter gene, *ADE2*, is inactive unless the gene is transactivated by p53. The p53-dependent *ADE2* gene was constructed in *S. cerevisiae* strains isogenic in every way except that a single promoter containing a unique response element drives transcription of the reporter gene in each strain.

**Figure 6:**
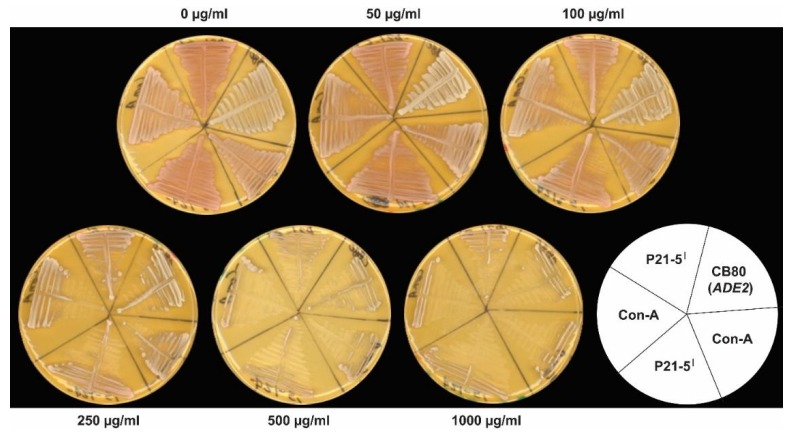
DNA-damage induced transactivation in *S. cerevisiae.* Isogenic strains yAFM containing the P21-5′ RE and yAFM containing the Con-A RE were used for the gene reporter assays. Both strains require adenine to grow, and the low amounts of adenine in yeast extract in non-synthetic complete (YPD) medium permitted their growth (ref. Materials and Methods). The plate layout of the strains is shown in the bottom right of Figure 6. On the YPD medium, yAFM containing the P21-5′ RE grows light red to pink in color, and yAFM containing the Con-A RE grows light pink. In each experiment, yAFM-P21-5′ and yAFM-Con-A were streaked in duplicate on each plate containing YPD medium alone and on YPD medium containing 50 µg/mL, 100 µg/mL, 250 µg/mL, 500 µg/mL, and 1000 µg/mL phleomycin D1. Drug concentrations in each plate are shown. An adenine-non-requiring strain (CB80) also was streaked and is white on all plates. Adenine-requiring (*ade2*) strains without either of the response elements (REs) were also used as controls in all experiments. The *ade2* strains were streaked in triplicate on separate plates in each experiment at each dose, and not squeezed onto the plates with strains bearing REs and the CB80 prototrophic control strain. The *ade2* strains grew red on YPD medium without phleomycin. They remained red and did not show any evidence of gene activation, (i.e., they never turned toward white) even at the highest doses, where growth was strongly inhibited. Moreover, no RE gene activation was observed in the *ade2* control strains after incubating the plates up to 10 days at room temperature or in the refrigerator. The results shown in Figure 6 are representative of four independent experiments.

We utilized this system, without introducing exogenous p53, to test for transactivation capabilities of a yeast protein or protein complex in response to DNA damage. We used phleomycin D1 to introduce DNA damage, including double-strand breaks, into cellular DNAs [40,66,67,68]. As described in the Figure 6 legend, one wild-type *ADE2* strain without the P21-5′ and Con-A RE’s, and two mutant *ade2* strains without the P21-5′ and Con-A RE’s, were used as controls in all experiments.

In the absence of phleomycin D1, the yAFM strain containing the P21-5′ RE was red, while the Con-A RE exhibited basal expression of the reporter since it grew lighter in color than the strain containing the P21-5′ RE (Figure 6). On 50 µg/mL phleomycin D1, the P21-5′ RE exhibited weak activity, similar to Con-A, and turned pink. We observed dose-dependent transactivation of the P21-5′ with increasing DNA damage from 50 to 250 µg/mL. This demonstrated that in the presence of DNA damage P21-5′-mediated gene expression was activated at a level comparable to the Con-A mediated response element. The *ADE2* control strain was white at all concentrations of phleomycin D1 (Figure 6), and the two mutant *ade2* control strains without RE’s remained red at all concentrations of phleomycin D1 (Figure 6 legend). All strains revealed dose-dependent growth inhibition or killing from 100 to 1000 µg/mL.

To check if survivors growing on phleomycin-containing media turned white because their mitochondria were nonfunctional [69], 1000 independent yAFM-Con-A and yAFM-P21-5′ white colonies on media containing 250 µg/mL to 1000 µg/mL phleomycin D1 were inoculated on YPD medium and YPG medium which inhibits the growth of cells that lose mitochondrial function. Only two colonies grew on YPD, but not YPG, medium, while the remaining 998 colonies grew well on both media. This frequency is in the range of the spontaneous loss of mitochondrial function (0.03% to 0.65%), during growth in the absence of phleomycin D1 [69], indicating white colonies growing in the presence of the drug typically retained mitochondrial function under these experimental conditions.

## 4. Discussions

These studies present the first evidence for a protein in *S. cerevisiae* that has increased capability after DNA damage of recognizing and binding specifically to known p53 binding sites. As described earlier, the addition of exogenous human p53 activates human p53-REs in yeast. Recently, it has even been shown that this exogenous p53-mediated activation can be inhibited following protein aggregation [70]. However, the ability of DNA damage to influence p53-RE activation activity in yeast had not been examined. We observed that an endogenous yeast factor not only demonstrated increased p53-RE DNA binding activity following DNA damage, but also was able to activate gene expression from a P21-5′ p53-RE. We observed a potentially significant affinity of Scp53BSF. Like mammalian p53 [47], the affinity of Scp53BSF for the SCS is higher than for the MDM2 oligonucleotide. The yeast Scp53BSF recognizes and binds specifically to the known p53 binding sites, and like mammalian p53, had elevated DNA binding activity in response to DNA damage. These studies also provide the first evidence for dose-dependent transactivation of a P21-5′ p53-RE reporter construct following drug treatment of yeast and supports previous work that no p53-RE transactivation occurs in yeast that has not incurred DNA damage. We examined the ability of proteins in yeast nuclear extracts to bind to the consensus and MDM2 p53 binding sites, and identified such a factor. The Scp53BSF appears to be the same size when bound to the SCS and natural MDM2 p53 binding sites. These results support a model for protein binding to p53 REs in yeast that works to activate transcription. Finding a protein or protein complex that recognizes and binds specifically to known p53 binding sites and finding protein activities that bind DNA and stimulate transcription are of great biological interest. Undoubtedly, this could open up new avenues of research.

The results suggest a sensing, or direct response, of Scp53BSF to DNA damage. Cells may use such a response signal to activate a DNA-damage checkpoint, or DNA-damage inducible repair, such as one we previously proposed is signaled by extensive chromosomal degradation [43]. The DNA-damage checkpoints at G1/S, intra-S, and G2/M stages of the cell cycle establish a link between DNA repair and cell cycle progression and can be referred to as intrinsic and extrinsic mechanisms. Extrinsic mechanisms are activated when DNA damage is detected, and these mechanisms, in turn, activate certain genes encoding proteins involved in DNA-repair pathways. The failure of these mechanisms can lead to malignancy and other disease conditions.

The Scp53BSF shows enhanced DNA binding activity after DNA damage, consistent with the situations in higher eucaryotes, where p53 is stabilized after DNA damage. Stationary-phase *S. cerevisiae* cells are growth-arrested and known to experience oxidative stress [71]. The results in this report suggest that when the cells are in the early stationary phase they are pressured to begin genomic surveillance. By analogy, levels of p53 are normally low in unstressed mammalian cells and are barely detectable by either gel shift assays or Western blot analyses [72].

Maintenance of genomic integrity following DNA damage is an active cellular response that enhances cell survival and limits heritable genetic abnormalities. Characterization of the biochemical steps in these signaling pathways should improve our understanding of (i) normal cell cycle regulation, (ii) mechanisms of cellular transformation following DNA damage, and (iii) responses of tumor cells and normal tissues to therapeutic radiotherapy and chemotherapy.

Based on the very well-characterized p53 DNA-binding activity with the synthetic and natural targets, we have found that the yeast nuclear protein extracts contain factors that can bind specifically to these p53 binding sites. The complexes that are formed might be due either to different oligomers of Scp53BSF bound to DNA or to other proteins combined with Scp53BSF.

In the future, identification and characterization of the components of the Scp53BSF are indispensable to further dissect comprehensively the pathways through which the complex functions to mediate the stress response. Using yeast as an excellent genetic system will facilitate studies that to date have been unable to be carried out because of the lack of facilitated genetic experiments. In addition, understanding how chemotherapeutic drugs signal to p53-independent pathways and cell cycle checkpoints remains of central relevance to cancer treatment, and using yeast to help dissect these pathways should help in targeted drug design.

The challenge to demonstrate that the protein(s) that recognize and bind specifically to the known p53 binding sites are the same one(s) that activate transcription can be met once the gene encoding Scp53BSF is in hand, or one or more subunits of the DNA-binding complex is identified. Screening for genes that encode the binding specificity and transcription activation functions is a reasonable approach in *S. cerevisiae*. The transactivation results reported here form the basis for a genetic screen for a deletion mutant no longer able to turn the *ADE2* reporter from red or pink to white. Finding such a deletion strain would suggest the activating factor was mutated. First, the Con-A construct could be introduced into a deletion mutant collection, and a candidate gene could be confirmed with a P21-5′ construct and EMSAs comparing isogenic strains with the wild-type and mutated factor. These and more future studies could help to determine targeted chemotherapeutic pathways that can be used when p53 is mutated.

Finally, we may have found in these studies an ancient p53-like DNA binding protein that shows activation after DNA damage signaling. The earliest p53 superfamily protein appears to have originated in single-celled Choanozoa, but no p53 superfamily member (p53, p63 or p73) has been found in fungi [73]. A p63-like domain structure appears to be evolutionarily ancient in some invertebrate p53 superfamily members, though not in fungi [73]. Thus, future studies could examine the interesting possibility that we have found an ancestor to the p53 superfamily members in *S. cerevisiae*.
